# A new method to analyse the pace of child development: Cox regression validated by a bootstrap resampling procedure

**DOI:** 10.1186/1471-2431-10-12

**Published:** 2010-03-05

**Authors:** Christian Denne, Sarah Maag, Nicole Heussen, Martin Häusler

**Affiliations:** 1 Department of Pediatrics, University Hospital RWTH Aachen, Pauwelsstrasse 30, 52074 Aachen, Germany; 2 Institute for Medical Statistics, University Hospital RWTH Aachen, Pauwelsstrasse 30, 52074 Aachen, Germany

## Abstract

**Background:**

Various perinatal factors influencing neuromotor development are known from cross sectional studies. Factors influencing the age at which distinct abilities are acquired are uncertain. We hypothesized that the Cox regression model might identify these factors.

**Methods:**

Neonates treated at Aachen University Hospital in 2000/2001 were identified retrospectively (n = 796). Outcome data, based on a structured interview, were available from 466 children, as were perinatal data. Factors possibly related to outcome were identified by bootstrap selection and then included into a multivariate Cox regression model. To evaluate if the parental assessment might change with the time elapsed since birth we studied five age cohorts of 163 normally developed children.

**Results:**

Birth weight, gestational age, congenital cardiac disease and periventricular leukomalacia were related to outcome in the multivariate analysis (p < 0.05). Analysis of the control cohorts revealed that the parents' assessment of the ability of bladder control is modified by the time elapsed since birth.

**Conclusions:**

Combined application of the bootstrap resampling procedure and multivariate Cox regression analysis effectively identifies perinatal factors influencing the age at which distinct abilities are acquired. These were similar as known from previous cross sectional studies. Retrospective data acquistion may lead to a bias because the parental memories change with time. This recommends applying this statistical approach in larger prospective trials.

## Background

During the past decades progress in intensive care medicine has markedly improved the prognosis of sick newborns. Despite this success, many children still face severe long-term medical educational and social problems as a result of a difficult perinatal period [1]. Numerous studies have focused on factors worsening neurodevelopmental outcome, for example identifying low APGAR scores, birth weight, umbilical artery pH and intraventricular hemorrhage (IVH) as infant-related factors as well as low social standing and drug abuse during pregnancy as maternal factors [2,3]. Most of these studies were cross sectional. None of them evaluated whether distinct risk factors influence the age at which distinct capabilities are acquired which would facilitate the detection and the promotion of children at risk for poor neurodevelopmental outcome.

The Cox regression model is employed to identify factors related to loss of function and patient survival. From a mathematical point of view, learning can be understood as loss of non-function which should allow using this model to identify factors that determine the age until which distinct capabilities will be aquired. Our group recently applied this approach to a small cohort of children with neonatal parenchymatous brain lesions. In this setting it proved useful, identifying unilateral versus bilateral neonatal parenchymatous brain lesion as prognostic factor [4]. In the framework of the Cox regression model the bootstrap resampling procedure is used for an investigation of stability and for the selection of factors included in a final model. This procedure involves sampling with replacement and refitting models on 100 distinct patient samples derived from the study population. The aim of the present study was to investigate if the combined application of the bootstrap resampling procedure for specification of candidate variables with a multivariate Cox regression analysis would prove appropriate to analyze a larger more heterogeneous group of children. The study is limited by its retrospective design, namely by the fact that the parents had to recall the ages until which different abilities had developed retrospectively. The ability to memorize these dates may differ from person to person and may interfere with the results. However, demonstrating the effectiveness of this statistical approach would justify the extensive efforts related to a large prospective trial.

## Methods

The primary study group consisted in all neonates (n = 796) admitted to the department of Pediatrics of RWTH Aachen University Hospital in 2000 and 2001. The parents of 447 of these 796 children agreed with a telephone interview and were questioned between 2005 and 2006 on the basis of a structured interview. The 447 children were included into further analysis as were data of the medical sheets of 19 children who had died during hospital stay within the first weeks of life.

The time interval of 4 to 6 years between birth and the parental interview was chosen because of two reasons: According to clinical experience, the majority of healthy children should have acquired all abilities defining the outcome variables. We also assumed that with increase of the follow-up interval the validity of our data might decrease, when parental memory becomes less accurate. Therefore we restricted the maximum time interval to 6 years and included a control group to evaluate this hypothesis. The control group consisted of 163 term-born children, correspondent to the number of eutrophic term-born study group children (n = 198). These children were selected from children admitted to our hospital because of acute infectious diseases, they had not been treated at our hospital in the neonatal period and were considered completely healthy by their parents. The children were only accepted if they had no neonatal complications and were separated in five age cohorts according to their year of birth (2000: n = 30, 2001: n = 31, 2002: n = 30, 2003: n = 49, 2004: n = 23).

The interview focused on the time of the acquisition of the milestones of development as were age in months of free sitting, standing, running and putting on a jacket, age of speaking single word and two-words-sentences, of bladder control, of eating with a spoon and of drinking with a cup. To provide a concise definition of influencing factors we preselected clearly-defined variables: maternal gestational diabetes and gestational hypertension, maternal alcohol or drug abuse, premature labor, gestational age, birth weight, 5-minute APGAR scores, umbilical cord pH, presence of neonatal cerebral hemorrhage, periventricular leukomalazia or birth asphyxia, congenital cardiac disease and newborn seizures. In consequence, not all of the various outcome factors known from the literature were included. For example, variables that are difficult to define retrospectively, such as neonatal infection, were omitted.

Clinical studies to identify perinatal complications had been performed adapted to the patients' risk profile which, however, should have detected the vast majority of patient problems. Cranial ultrasound for example was restricted to preterm children and term children with assumed high risk for brain lesions such as asphyxia or vacuum extraction applying the classification proposed by Papile et al. [5], and cardiac ultrasound was performed in children showing clinical symptoms for congenital cardiac disease.

Age was adjusted for prematurity by calculating the estimated date of birth from the assessment of gestational age to arrive at a birth date based on an expected 40 weeks' gestation.

For selection of important factors that might be related to neonatal outcome we used a procedure proposed by Sauerbrei et al. [6]. With regard to the retrospective study design we chose strategy A, recommended for the selection of weak factors. First 100 bootstrap samples were generated. Then a multivariate Cox regression model with backward selection was fitted to each replication of the original data set and for each ability. The significance level for removing a factor from the model was set to 0.05. Subsequently the relative frequency for each factor to be included in the model was computed. Factors selected at a frequency of 30% or more were then analyzed by means of chi-square test to prove independence of the inclusion frequencies. A factor for which independence in combination with all others could not be rejected was included in a final multivariate Cox regression model. In case that a pair of factors was found dependent, the factor showing a lower conditional and unconditional inclusion frequency was eliminated from the final model. For all Cox models, graphical and numerical methods according to Lin et al. were performed to establish the validity of the proportionality assumption [7]. No deviation from model assumption could be observed.

To address a possible bias of the retrospective study design we investigated the influence of the time elapsed since birth on parents' assessment of abilities by univariate Cox regression.

As all statistical tests were conducted solely in an explorative manner, no α-adjustment for multiple testing was carried out [8]. Thus, p-values of p ≤ 0.05 could be interpreted as statistically significant test results with respect to the investigated collective of this study. The analysis was performed with the Statistical Package for Social Sciences 11.0 (SPSS Inc., Chicago, Il, USA) and with the SAS Release 9.1.3 (SAS Institute Inc., Cary, NC, USA).

The follow-up data on neonates treated at RWTH Aachen University Hospital have been collected in the course of the quality assurance program for level three perinatal centers (according to the fifth german federal social law book (Sozialgesetzbuch, SGB V) §137, paragraph 1, section 3, No.2). During the preparation process the necessity for an additional ethics vote was carefully discussed within the study group and with the leading authorities of RWTH Aachen University Children's Hospital. Considering the fact that no child was actively or directly involved in this retrospective questionnaire-based investigation and in line with the regulations on the requirements for ethical approval at RWTH Aachen University Hospital in Germany, it was agreed that no ethics vote would be necessary.

## Results

The bootstrap procedure identified various candidate factors possibly related to neonatal outcome, including premature labor, maternal gestational diabetes and hypertension, periventricular leukomalazia (PVL), congenital cardiac disease, gestational age, birth weight, 5-minute APGAR scores, the presence of cerebral hemorrhage, newborn seizures, the umbilical cord pH and perinatal asphyxia (Additional file 1).

**Figure 1:**
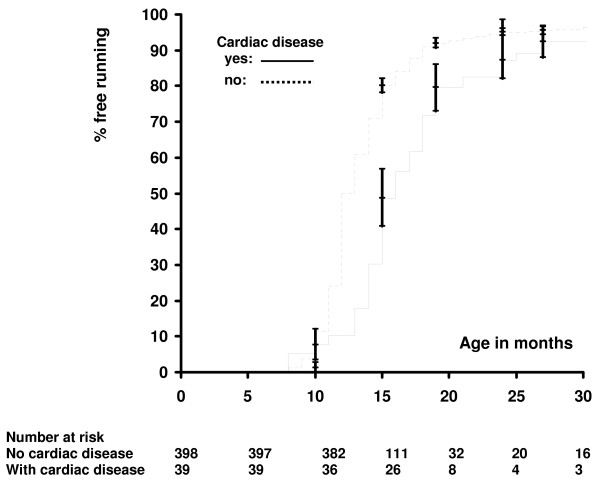
**Kaplan-Meier plot for the acquisition of free running in children with congenital cardiac disease**. The acquisition of free running is delayed in children with congenital cardiac disease (median time: 16 months. 95% confidence interval: 15;18 months. Interquartile range: 5 months) compared with children without (median time: 12 months. 95% confidence interval: 12;13 months. Interquartile range: 4 months). Vertical bars indicate standard errors. No censored cases were observed within 30 months of follow-up.

The subsequent multivariate Cox analysis revealed four of these variables to be independently related to distinct outcome parameters (Additional file 2): Low birth weight was related to delayed ability of using a spoon, gestational age was related to a delayed acquisition of bladder control during night and days, the presence of PVL was related to a delay in free sitting, free standing and free running, and the presence of congenital cardiac disease was related to a delay in free standing and free running. Additional file 3 displays the respective hazard ratios and corresponding 95% confidence intervals. To illustrate the relationship between the presence of congenital cardiac disease and the acquisition of free running a Kaplan-Meier plot was performed for this variable (Figure 1).

Control group cohorts were studied to find out whether the time interval between the date of achieving a distinct ability and the interview date might influence the parents' assessment. The different cohorts varied statistically significantly with regard to the variables bed wetting at day and bed wetting at night and days (Additional file 4). The more time after birth elapsed, the parents suggested an earlier acquisition of the respective ability.

## Discussion

Impaired neuromotor development is of profound medical and socioeconomic impact. The most serious problems may arise in preterm infants born before 30 completed weeks of gestational age. Identification of factors related to poorer outcome can contribute to ameliorate perinatal maternal and pediatric therapy, to improve the prognosis and to facilitate treatment of children at risk for severe disabilities.

Outcome studies to identify factors contributing to impaired neuromotor development classically show a cross sectional design which compares the abilites of different patient groups at a distinct age after birth. Different patient groups, however, might additionally differ with regard to the speed by which a distinct capability is acquired. For example, when looking at Figure 1 no difference in outcome would be noted in a cross sectional approach when studying early (10 months) or late (30 months) after birth, respectively, whereas a significant difference can be expected after 15-20 months. Therefore, in cross sectional studies, the time interval seems critical with regard to obtain positive results. This problem is reduced when applying the Cox regression model, enabling to focus on differences related to the speed by which distinct capabilities are acquired. In this process, identification of candidate factors to be included into the multivariate Cox regression model is a critical point. This is frequently based on a univariate Cox regression analysis or selection procedures as stepwise, forward or backward selection, resulting in a single model without information about its stability. In contrast, the bootstrap resampling procedure used in the here reported study is considered to estimate the whole distribution of importance for the factors under consideration [6].

Combined application of the bootstrap resampling procedure and the multivariate Cox regression analysis identified four variables significantly related to neonatal outcome: birth weight, gestational age, the presence of PVL and of congenital cardiac disease. This result per se does not prove that these factors might also be clinically relevant. However, all these variables have been considered of clinical importance in previous studies which strongly underlines the diagnostic impact of this statistical approach, which has not been reported previously for analysis of neonatal outcome.

Not all prognostic factors reported in literature, as were maternal substance (drugs, alcohol, nicotine) abuse in pregnancy[9], gestational diabetes[10], newborn seizures[11] or asphyxia proved significant in the present study [12]. However, considering the limited number of children in general as well as the relatively small patient groups it seems encouraging that the combined application of the bootstrap resampling procedure and the Cox regression model identified such a large number of significant factors. For example, the presence of congenital cardiac disease was identified as a significant factor. Among the here reported patients the calculated median time for patients with and without congenital cardiac disease to acquire the ability of free walking was 16 and 12 months, respectively. In this setting assuming an accrual period of 24 months and a maximum follow-up time of 48 months, a sample size per group of 230, with a total number of 376 events is required to obtain a power of at least 80% in an exponential maximum likelihood test of equality of survival curves with a 5% two-sided significance level. If the follow-up time will be extended the required sample size will be reduced. For example, a follow-up time of 96 months, leads to a sample size of 192 per group.

To assess the impact of a combined bootstrap/Cox regression approach we have also performed Cox regression analysis without a preceding bootstrap resampling procedure (data not shown) which could identify all variables identified during the combined approach However, 14 additional variables which differed from those identified during the bootstrap resampling procedure were also identified but not attributed a lower impact which underlines the superiority of the combined bootstrap/Cox regression over the isolated Cox regression approach.

A major limitation of the present study was its retrospective design. For example, analysis of the healthy control group clearly revealed that the parental memory on their children's development changes the more time after birth has passed, which became obvious concerning the variables no bedwetting all day long respectively no bedwetting only at day. This strongly recommends to analyse neonatal outcome in prospective study designs which may then include bootstrap strategy B, as outlined by Sauerbrei et al. [6] This strategy is mainly based on the application of higher inclusion frequencies than used in strategy A and may lead to the detection of stronger factors.

## Conclusions

Combined application of the bootstrap resampling procedure and multivariate Cox regression analysis effectively identified perinatal factors influencing the speed of learning. These were similar as known from previous cross sectional studies. Retrospective data acquisition leads to a bias because of parental memories change with time. This problem does not question the statistical procedure per se. However, considering the clinical consequences of under- respectively overestimating the impact of distinct perinatal conditions, prospective studies must be performed to clearly specify the factors that determine the speed of pediatric neuromotor development.

## Competing interests

The authors declare that they have no competing interests.

## Authors' contributions

CD participated in data collection, data analysis and preparation of the manuscript. SM participated in the data collection and data analysis. NH provided the statistical background knowledge and revised the manuscript. MH contributed to writing the manuscript and coordinated the study. All authors read and approved the final manuscript.

## Pre-publication history

The pre-publication history for this paper can be accessed here:

http://www.biomedcentral.com/1471-2431/10/12/prepub

## Supplementary Material

Additional file 1 **Table 1**. Variables identified during the bootstrap resampling procedure and their inclusion frequencies. Click here for file

Additional file 2 **Table 2**. p-values obtained during the multivariate Cox regression analysis using variables selected during the bootstrap resampling procedure. Click here for file

Additional file 3 **Table 3**. Hazard ratios, 95% confidence intervals of factors found significant in the multivariate Cox regression analysis. Click here for file

Additional file 4 **Table 4**. Comparison of five control cohorts among each other by univariate Cox regression analysis (years of birth from 2000 to 2004, n = 163). Click here for file
