# Supplementary material for: A new method to analyse the pace of child development: Cox regression validated by a bootstrap resampling procedure
Source: BMC Pediatr. 2010 Mar 5;10:12. doi: 10.1186/1471-2431-10-12 (PMC2837865; doi:10.1186/1471-2431-10-12)
Supplement: Additional file 1 — Table 1. Variables identified during the bootstrap resampling procedure and their inclusion frequencies. [file 1471-2431-10-12-S1.PDF]

Table 1: Variables identified during the bootstrap resampling procedure and their inclusion frequencies.

	Free sitting	Free standing	Free running	Putting on a jacket	Speaking single words	Speaking word combinations	Drinking out of a cup	Using a spoon	No bed-wetting at day	No bed- wetting at night and days
Premature labor	**35**	34*	36*	6	**43**	**50**	**34**	38*	6	5
Gestational diabetes	6	5	5	12	**30**	16	5	5	13	**49**
Gestational age	18	11	15	27	15	16	29	**43**	18	**38**
Birth weight	**39**	5	9	28	9	14	**31**	**37**	14	22
5-minute APGAR score	16	16	15	11	22	**35**	9	12	12	14
Cerebral hemorrage	16	4	13	8	2	13	**32**	20	4	0
Periventricular leukomalacia	**48**	**33**	**30**	12	2	15	4	10	3	0
Congenital cardiac disease	7	**37**	**50**	**44**	19	19	5	20	12	38*
Newborn seizures	20	7	13	**30**	20	0	6	**30**	3	14
Gestational hypertension	10	3	3	18	18	**55**	5	11	5	9
Maternal drug abusus	0	1	1	11	2	8	1	2	1	2
Maternal alcohol abusus	0	0	0	0	0	0	0	0	0	0
Maternal nicotine abusus	6	7	21	3	2	12	4	7	12	0
Low pH of umbilical cord	4	8	9	28	13	2	4	8	3	**32**
Asphyxia	11	17	17	**42**	7	2	5	8	4	**39**

Total study group n=466.

IVH= intraventricular hemorrhage; PVL= periventricular leukomalacia. Significant findings are highlighted.

* eliminated in step 4 of the bootstrap approach
