# Supplementary material for: A new method to analyse the pace of child development: Cox regression validated by a bootstrap resampling procedure
Source: BMC Pediatr. 2010 Mar 5;10:12. doi: 10.1186/1471-2431-10-12 (PMC2837865; doi:10.1186/1471-2431-10-12)
Supplement: Additional file 2 — Table 2. p-values obtained during the multivariate Cox regression analysis using variables selected during the bootstrap resampling procedure. [file 1471-2431-10-12-S2.PDF]

Table 2: p-values obtained during the multivariate Cox regression analysis using variables selected during the bootstrap resampling procedure.

	Free sitting	Free standing	Free running	Putting on a jacket	Speaking single words	Speaking word combinations	Drinking out ofa cup	Using a spoon	No bed wetting at night and days
Premature labor	0.3073				0.0976	0.3390	0.2577		
Gestational diabetes					0.4823				0.1239
Gestational age								0.2767	**0.0230**
Birth weight	0.0993						0.2444	**0.0332**	
5-minute APGAR score						0.2162			
Cerebral hemorrage							0.8848		
Periventricular leukomalacia	**0.0218**	**0.0420**	**0.0326**						
Congenital cardiac disease		**0.0199**	**0.0011**	0.2983					
Newborn seizures				0.8420				0.2250	
Gestational hypertension						0.3959			
Low pH of umbilical cord									0.4629
Asphyxia				0.7483					0.6367
Number of cases available (censored)	442 (7)	435 (12)	437 (13)	400 (28)	442 (5)	394 (13)	302 (8)	432 (12)	335 (106)

Factors proven significant ($p < 0.05$) during the multivariate Cox regression analysis are highlighted.
The factors, maternal abuse of nicotine, alcohol or drugs, as well as bed wetting at day are no longer displayed as these were not included into the multivariate Cox regression analysis.
