# Supplementary material for: A new method to analyse the pace of child development: Cox regression validated by a bootstrap resampling procedure
Source: BMC Pediatr. 2010 Mar 5;10:12. doi: 10.1186/1471-2431-10-12 (PMC2837865; doi:10.1186/1471-2431-10-12)
Supplement: Additional file 3 — Table 3. Hazard ratios, 95% confidence intervals of factors found significant in the multivariate Cox regression analysis. [file 1471-2431-10-12-S3.PDF]

Table 3: Hazard ratios, 95% confidence intervals of factors found significant in the multivariate Cox regression analysis.

Outcome variable	Factor	Hazard ratio	95% confidence interval
Free sitting	Periventricular leukomalacia	0.261	[0.083 ; 0.823]
Free standing	Periventricular leukomalacia	0.307	[0.098 ; 0.958]
	Congenital cardiac disease	0.669	[0.476 ; 0.938]
Free running	Periventricular leukomalacia	0.289	[0.092 ; 0.902]
	Congenital cardiac disease	0.570	[0.406 ; 0.800]
Using a spoon	Birth weight		
	< 1500g	0.303	[0.133 ; 0.692]
	1501-2000 g	0.590	[0.313 ; 1.111]
	2001-2500 g	0.940	[0.701 ; 1.262]
Bed wetting at night and days	Gestational age		
	24-29 completed weeks	0.453	[0.264 ; 0.778]
	30-32 completed weeks	0.970	[0.563 ; 1.676]
	33-36 completed weeks	1.081	[0.820 , 1.442]
