# Supplementary material for: A new method to analyse the pace of child development: Cox regression validated by a bootstrap resampling procedure
Source: BMC Pediatr. 2010 Mar 5;10:12. doi: 10.1186/1471-2431-10-12 (PMC2837865; doi:10.1186/1471-2431-10-12)
Supplement: Additional file 4 — Table 4. Comparison of five control cohorts among each other by univariate Cox regression analysis (years of birth from 2000 to 2004, n = 163). [file 1471-2431-10-12-S4.PDF]

Table 4: Comparison of five control cohorts among each other by univariate Cox regression analysis (years of birth from 2000 to 2004, n=163).

	No bedwetting at night and days	No bedwetting at day
Hazard ratio	0.790	0.649
95% confidence interval	[0.644 ; 0.971]	[0.489 ; 0.861]
Probability according to Wald statistics	0.025	0.003
